# Developing a Framework for Self-regulatory Governance in Healthcare AI Research: Insights from South Korea

**DOI:** 10.1007/s41649-024-00281-w

**Published:** 2024-03-25

**Authors:** Junhewk Kim, So Yoon Kim, Eun-Ae Kim, Jin-Ah Sim, Yuri Lee, Hannah Kim

**Affiliations:** 1 Department of Dental Education, College of Dentistry, Yonsei University, Seoul, South Korea; 2 Asian Institute for Bioethics and Health Law, Department of Medical Humanities and Social Sciences, College of Medicine, Yonsei University, Seoul, South Korea; 3 Center for Research Compliance, Ewha Womans University, Seoul, South Korea; 4 Department of AI Convergence, Hallym University, Chuncheon, South Korea; 5 Department of Health and Medical Information, Myongji College, Seoul, South Korea

**Keywords:** Healthcare AI research, Research ethics, Research governance, AI governance, Self-regulation, Interdisciplinary approach

## Abstract

This paper elucidates and rationalizes the ethical governance system for healthcare AI research, as outlined in the ‘Research Ethics Guidelines for AI Researchers in Healthcare’ published by the South Korean government in August 2023. In developing the guidelines, a four-phase clinical trial process was expanded to six stages for healthcare AI research: preliminary ethics review (stage 1); creating datasets (stage 2); model development (stage 3); training, validation, and evaluation (stage 4); application (stage 5); and post-deployment monitoring (stage 6). Researchers identified similarities between clinical trials and healthcare AI research, particularly in research subjects, management and regulations, and application of research results. In the step-by-step articulation of ethical requirements, this similarity benefits from a reliable and flexible use of existing research ethics governance resources, research management, and regulatory functions. In contrast to clinical trials, this procedural approach to healthcare AI research governance effectively highlights the distinct characteristics of healthcare AI research in research and development process, evaluation of results, and modifiability of findings. The model exhibits limitations, primarily in its reliance on self-regulation and lack of clear delineation of responsibilities. While formulated through multidisciplinary deliberations, its application in the research field remains untested. To overcome the limitations, the researchers’ ongoing efforts for educating AI researchers and public and the revision of the guidelines are expected to contribute to establish an ethical research governance framework for healthcare AI research in the South Korean context in the future.

## Introduction

The rapid progress of machine learning and artificial intelligence (AI) poses new and unprecedented challenges to the entire healthcare sector. Particularly, as a critical extension of the foundational discussions on the technology adoption in healthcare (Rajpurkjar et al. 2022), the focus now shifts towards the practical governance and regulation of AI development and its application in healthcare landscape. South Korea has swiftly embraced biomedical technologies, showcasing a clear inclination in integrating AI in healthcare. The ‘2022 Medical Device License Report’ from the Ministry of Food and Drug Safety (MFDS) of the Republic of Korea unveils that a total of 149 AI-based medical devices obtained approval and certification in the country, with 10 receiving approval and 38 attaining certification in 2022 (MFDS 2023).

Corresponding with this trend, the Korean National Institutes of Health (KNIH) published the ‘Research Ethics Guidelines for AI Researchers in Healthcare’ in August 2023, marking an initial effort to offer an actionable guidance to healthcare AI researchers in the country (KNIH 2023). The guidelines aim to establish ethical standards for all stages of healthcare AI development by presenting ethical principles and detailed values. The researchers mainly participated in developing the guidelines using robust research methodologies, such as literature reviews, interdisciplinary consultations, and a public hearing as well as providing empirical research evidence from surveys for the lay public and experts. Consequently, the guidelines present six principles with corresponding codes and explanations. The principles, stemmed from the World Health Organization (WHO) report ‘Ethics and governance of artificial intelligence for health’, are tailored for the national context, providing a framework for researchers to evaluate their research practices. Importantly, it is noted that while bioscientists are well-versed in the ethical procedures and legal regulations related to human subjects research, those in computer science and data science engaged in healthcare AI research may lack familiarity with these standards (Metcalf and Crawford 2016; Throne 2022). Consequently, these guidelines are designed to assist support healthcare AI researchers in conducting ethical research by presenting providing part I principles to consider in relation to research, part II corresponding relevant research codes, regulations and related ethical cases, and part III an expanded framework, aligning with that applies for the existing governance framework for phase I–IV clinical research, tailored for to the context of healthcare AI research.

The purpose of this paper is to outline and provide rationales for the ethical governance system introduced in the part III of the guidelines. At present, we are in the process of translating the guidelines for an official English version. Amidst this ongoing endeavour, this paper preliminarily introduces the final section of the guidelines, which is under linguistic review. Subsequently, we describe the governance framework, comprising six steps, accompanied by ethical and institutional explanations for each stage. In conclusion, this paper presents a healthcare AI research governance system, expanding upon the existing human subjects research. It advocates for the establishment of a robust, secure, and sustainable research governance structure by adapting the clinical research system prevalent in countries where such approaches are already established to the domain of healthcare AI research governance.

## Procedural Considerations for Conducting Healthcare AI Research

Aforementioned, part I of the guidelines provide background, developing process and methodologies, aims, scope, and key terms. Next, part II reviews the existing legal frameworks related to safety and effectiveness, liability for errors and negligence, privacy laws for patient data protection, and legal frameworks responding to bias and discrimination. Based on the legal background, part III introduces a six principle-based framework and explanations with specific ethical cases, aligning with the procedural considerations when researchers conduct healthcare AI research.

Particularly, part III of the guidelines is grounded in six ethical principles: (a) respect for and protection of human autonomy; (b) promotion of human well-being, safety, and the public interest; (c) ensuring transparency, explainability, and reliability; (d) upholding accountability and legal obligations; (e) promoting inclusivity and equity; and (f) fostering responsiveness and sustainability (Kim et al. 2023). While these principles align with those of the WHO, specific codes and applications have been tailored to suit the national context. The healthcare AI research governance framework presented herein also follows this approach, incorporating relevant principles to be considered at each stage.

The guidelines restructured the principles by the steps of the research process as a form of a checklist. This checklist provides a baseline for all stakeholders the field to voluntarily identify and assess the ethical considerations pertinent to practical research and development (Table 1).

**Table 1:** Ethical considerations at each stage of healthcare AI research and development

Stage	Requisites	Reviewers	Related principles
1. Preliminary ethics review	Self-designed ethics framework based on the guidelines	Researchers and developers, research institutions	Respect and protect human autonomy Promoting human happiness, safety, and the public interest Transparency, explainability, and reliability Accountability and legal obligations Inclusivity and equity Responsiveness and sustainability
2. Creating datasets	Data management protocols Data Review Board (DRB) review	Data Creator, DRB	Respect and protect human autonomy Inclusivity and equity Responsiveness and sustainability
3. Model development	Set-up algorithms Model development plans Institutional Review Board (IRB) review	Researchers and developers, Research institutions, IRB	Transparency, explainability, and reliability Inclusivity and equity
4. Training, validation, and evaluation	Model training and testing Internal validation	Researchers and developers, Research institutions	Transparency, explainability, and reliability Accountability and legal obligations Inclusivity and equity
5. Application	External validation Compliance with ethical and legal regulations	Researchers and developers, Government	Respect and protect human autonomy Transparency, explainability, and reliability Accountability and legal obligations
6. Post-deployment monitoring	Open communications Continuous monitoring through feedbacks	Researchers and developers, Suppliers, Users, Government	Respect and protect human autonomy Promoting human happiness, safety, and the public interest Transparency, explainability, and reliability Accountability and legal obligations Inclusivity and equity Responsiveness and sustainability

Healthcare AI research and development begins with the establishment of a robust ethical framework, grounded in the aforementioned six ethical principles. A multidisciplinary team collaborated to establish the ethical considerations for research and development and delineate the requisite compliance measures. The AI development process comprises distinct stages: data collection, algorithm development, model training integration, and evaluation. Each steps follows a structured ethical framework, integrating the principles, thereby ensuring the ethical integrity of healthcare AI research and development. Periodic evaluations are conducted to assess ethical compliance and identify areas for improvement. Furthermore, continuous feedback is sought following the application of the developed model in real-world environment.

For research institutions, the guidelines play a pivotal role in ensuring ethical standards of healthcare AI research and development. The research institutions can utilize the guidelines to evaluate the design procedure, algorithm development, and application of AI technologies in their own research endeavours. This assessment entails evaluating the alignment of the guidelines with domestic laws, international norms, and societal dialogues. Additionally, it is advisable for review committees and institutions that oversights healthcare AI research and development to implement reasonable and responsible regulations to manage research activities, educating and informing stakeholders about these regulations, and maintaining open communication for ongoing revisions and amendments as required.

Furthermore, through such feedback and societal discussion, the developers of this guidelines strive for continuous refinement, aiming to foster a research environment that esteems ethical principles and values.

## Stage 1. Preliminary Ethics Review

Prior to the commencement of healthcare AI research and development, it is imperative to establish a clear ethical framework guided by specific guidelines. This preliminary stage is the responsibility of the organization, tasked with laying the foundational groundwork. They should actively seek advice through public participation action from a diverse array of stakeholders, including patients, the public, and expert groups such as medical ethicists and legal scholars, to ensure a well-rounded perspective through public participation action. Additionally, it is essential to establish and consider ethical guidelines that are particularly relevant to the research and development process, setting a strong foundation for responsible and ethical AI innovation in healthcare.

Related questions:

- Does the plan include sensitive objectives? Is the objective to develop a medical device or other health and public health objectives? (Specify clinical diagnosis-treatment decision, patient decision support, prevention, behavioural intervention, public health, and if others, additional descriptions should be included in the protocol.)
- Is it human subject research or research utilizing datasets? (check bioethics exemptions and compliance requirements.) If human subjects research, does the plan include interventions or interactions?
- Does the plan address potential or manifest harms? (Provide a risk-benefit analysis.)
- Is there evidence or potential for sample bias in the plan?

## Stage 2. Creating Datasets

In the process of collecting and processing data for healthcare AI model development, several key considerations must be addressed. Initially, it is essential to evaluate the collectability, availability, and intended use of the data. Depending on the potential risk for privacy infringement, appropriate measures such as anonymization or pseudonymization should be employed for the dataset. A detailed data collection plan is crucial to outline the methods and objectives clearly. Additionally, conducting ongoing quality control is imperative to minimize data bias and ensure the diversity and representativeness of the datasets, which are fundamental for the development of fair and effective healthcare AI systems.

Related questions:

- Is the data collection plan comprehensive? (identification and consultation with data subjects or maintaining organizations, data types and details, collection techniques, frequency selection, inclusion and appropriateness of purposes of use)
- Are anonymization measures considered? (detailed technical and administrative/physical measures; if not anonymized, justification and additional measures required)
- Is the dataset size aligned with the learning task and model complexity?
- Is the data quality recognized as high?
- Are the data appropriately visualized and exploratory analyses conducted?
- Is the raw data collected according to approved clinical standards and protocols, utilizing valid and reliable techniques?
- Are regular and continuous data quality control measures implemented?

## Stage 3. Model Development

Configuring algorithms to align with research objectives and applying preliminary data to assess appropriateness is a critical phase in AI development. Developers should build the model using decision-making algorithms aimed at achieving specific, predefined goals. To ensure transparency, a concise description of the development plan should be publicized, detailing the steps and intentions behind the model’s construction. Standardizing the data before training the model is essential to ensure consistency and accuracy. Additionally, it is crucial to specify any methodological considerations that might reveal bias within the dataset, thereby allowing for adjustments and improvements to maintain integrity and fairness in the model's outcomes.

Related question (considerations in Stage 1 should be considered in conjunction with those below)

- Does the plan provide an adequate accounting of human subjects and data subjects?
- Are the methods of split cross-validation of datasets and datasets utilized in the plan appropriate? (correcting erroneous data, resolving inconsistencies in data, deleting unnecessary data, ensuring quality assurance and accuracy of data)
- Are potential issues with privacy addressed? (review for possible data breach)
- Does the plan assess the sources or likelihood of sampling/evaluation/algorithmic bias? (considering resampling, algorithmic fairness, etc.)

## Stage 4. Training, Validation, and Evaluation

The phase of training and validating algorithms using the collected data, followed by an evaluation of their applicability for research purposes, is crucial for crafting robust AI systems. Training AI models meticulously is fundamental to boost their reliability and accuracy. It is also critical to ensure that the AI models undergo thorough internal validation through appropriate procedures to confirm its effectiveness and safety in practical applications. Moreover, implementing measures to assess clinical reliability is necessary for healthcare AI development. This includes evaluating the AI’s accuracy, its relevance to clinical applications, the fairness of its decision-making processes, and the level of trust or acceptance these systems receive from both patients and healthcare professionals.

Related questions:

- Does the model use a transparent methodology for AI data mining and project implementation? (e.g., CRISP-DM,^1^ KDD,^2^ SEMMA,^3^ CPMAI^4^)
- What is the model’s purpose? (specify predictive models, text mining, automation, record abstraction, biometrics, and if others, additional descriptions should be in the protocol)
- What kind of technology is utilized? (specify machine learning, deep learning, natural language processing, unsupervised learning, reinforcement learning, and if others, additional descriptions should be included in the protocol.)
- Can any unexpected results be analysed or tracked?

## Stage 5. Application

Ensuring compliance with ethical frameworks and legal regulations is paramount when governing AI models in the real-world application. AI models functioning as medical devices, tasked with analysing data for disease diagnosis, management, and prediction, must comply with approval and review protocols established by relevant regulatory bodies. Those covered by health insurance require safety, effectiveness, and economic evaluations by designated authorities. Implementing an external validation process that involves public participation can further reinforce the model’s integrity and social acceptance.

Furthermore, it becomes crucial that clinical AI algorithms to prioritize user-friendliness, requiring minimal training to lessen cognitive load and streamline decision-making. Supervising and maintaining the models involve assessing their ethical integrity and making continuous improvements as necessary. Clearly designate a specific individual or entity responsible for the ethical management of the model.

Related questions:

- Is there a match between the dataset and the population setting for model application?
- Are the results interpretable?
- Have they been assessed for major biases? (e.g., gender, race)
- Has the model been externally validated using datasets from other settings?
- Has the model been empirically evaluated for validity, clinical utility, and cost-effectiveness?

## Stage 6. Post-deployment Monitoring

Continuing engagement with model users and refining the model based on their feedback is essential in this stage. It involves regularly reviewing the model’s performance in real-world applications, aligning with the self-constructed ethical framework previously established. Maintaining open communication and collaboration with all stakeholders, including AI providers, users, patients, the public, and government agencies, is crucial for ongoing development and alignment with user needs and ethical standards. Furthermore, ensuring that the models can be seamlessly integrated into existing production environments is vital for effective decision-making based on real data. This stage emphasizes the importance of adaptability and responsiveness to the evolving landscape of AI applications and societal impacts.

Related questions:

- Do you regularly monitor the product whether the entire data process is correctly aligned or when the entire process is performed automatically without the need for human intervention?
- Does the user (healthcare provider), user organization (healthcare organization) regularly disclose usage results, both positive and negative?
- Are there communication and recovery protocols established for model application errors?
- Are there improvements needed in the relevant ethical framework and guidelines?

## A Step-by-Step Explanation of Healthcare AI Research Governance Framework

The healthcare AI research governance framework delineated above adapts and extends the phase I–IV process for human clinical research to healthcare AI research. This adaptation allows guideline developers to manage and regulate research more reliably by extending existing research governance procedures, thus reducing the need for designing new schema for healthcare AI research ethics. This approach reduces training efforts and provides a foundation for researchers to quickly comprehend and apply the governance framework. Additionally, many of the administrative resources already established for human subjects research can be leveraged for healthcare AI research.

However, it is imperative to analyse the commonalities and divergences between clinical trials and healthcare AI research. This paper presents the similarities in terms of (a) research subjects, (b) areas of research management and regulation, and (c) application of research results. On the other hand, there are differences between clinical trials and healthcare AI research, including (a) the research and development process, (b) evaluation of research results, and (c) the modifiability of research results.

Firstly, human subjects, biospecimens, or populations in clinical trials share qualitative similarity with health data, their constructs, or databases utilized in healthcare AI research. For instance, biospecimens are recognized for their uniqueness—characteristics derived from the individuals they originate from—and then, health data collected from human subjects possess the same ontological nature as derivatives of individuals. They inherently refer to persons and are intricately connected to them (Cha and Kim 2022). Health datasets encapsulate various biological, behavioural, and socioeconomic records of a specific data subject, directly linked with the human body. The linkage of whole genome sequencing (WGS) data to personal identity intertwines the human body with the data presenting (Li et al. 2014). In population studies, the population database reflects the target population group, and eventually, they should become ontologically and practically identical.

Secondly, both clinical trials and healthcare AI research aim to derive results that benefit humans—whether it is treatments, new drugs, medical technologies, and biomaterials in clinical trials, or algorithms and applications in healthcare AI research. Just as clinical research with human subjects has established protocols to ensure respect and protection of individuals involved and affected by research process and its outcome (National Commission for the Protection of Human Subjects of Biomedical & Behavioral Research 1978), healthcare AI research also confronts to address ethical considerations arising from both the research process and the utilization of its outcomes. The considerations encompass aspects ranging from the respecting and protection of individuals to issue of accountability and sustainability. Similar to the human subjects research oversight by Institutional Review Boards (IRBs), which review and monitor all biomedical research, healthcare AI research necessitates a robust review and monitoring process. This process is crucial even when certain research activities might be exempt from regulatory requirements, acknowledging the unique challenges and potential risks associated with AI. A tailored oversight mechanism for healthcare AI is imperative that all research involving human subjects—or their data—is conducted responsibly and ethically. As human clinical trials aim to apply developed treatments and new drugs to humans by assessing efficacy and safety, healthcare AI research endeavours to apply developed algorithms and applications to humans to demonstrate effectiveness.

Recognizing the identified similarities, it could be argued that the governance framework established for human clinical research can be directly applied to healthcare AI research. However, significant differences between human clinical research and healthcare AI research necessitate a tailored approach.

Primarily, a distinction lies in the development process between human clinical research and healthcare AI research. Human clinical research focuses on developing of treatments or new drugs, validated through assessments of safety and effectiveness and comparative benefit analyses. Upon affirming these steps, a treatment or drug is considered developed, thereafter maintained through post-marketing/application monitoring or management. Conversely, healthcare AI research entails an iterative process of development, refinement, and validation of algorithms or applications, inherently characterized by their modifiability (Higgins and Madai 2020). This research paradigm encompasses a series of stages from data collection to algorithm application and continual revision through feedback loops. Throughout the progress, algorithms are expected to continuously learn, revise, and evolve (Pianykh et al. 2020). Therefore, a governance approach tailored to this process, spanning from data collection and algorithm development to model training integration, and evaluation becomes essential.

The primary difference consequently leads to variations in how research outcomes are evaluated and modified. Clinical trials typically employ statistical validation methods like randomized controlled trials (RCTs) or equivalent methodologies to confirm effectiveness. In contrast, healthcare AI research assesses performance using metrics such as the area under the receiver operating characteristic (ROC) curve (AUC) derived from collected data (Wu et al. 2021), which involves trade-offs between false positives and false negatives. In addition, drugs and medical devices approved through clinical trials are subject to re-evaluation if modifications are made. However, in healthcare AI research, accepting modifications poses a challenge due to its continuous learning nature, disrupting the notion of a consistent “product-based view” (Gerke et al. 2020). Therefore, it is practical for governing healthcare AI research governance to consider adopting elements maintainable from the human subjects clinical research governance system while modifying them to suit the development and application dynamics of healthcare AI.

## Six-Stage Process for Healthcare AI Research

Given these considerations, this guidance extends the traditional four-phase clinical research process (phase I: safety; phase II: efficacy and side-effects; phase III: large trials; phase IV: post-market surveillance) by introducing a six-stage process for healthcare AI research. The introduction of <Stage 1: preliminary ethics review > and < Stage 2: creating datasets > reflects the unique nature of healthcare AI research and emphasizes the necessity for comprehensive and sustainable research guidelines from data collection stage onwards. < Stage 3: model development > , < Stage 4: training, validation, and evaluation > , < Stage 5: application > , and < Stage 6: post-deployment monitoring > align with the concepts of phases I–IV of clinical research but are specifically tailored to address the characterized process of developing and applying healthcare AI algorithms.

- **Stage 1** necessitates researchers and developers to establish an ethical framework tailored to their research objectives. This endeavour enables the research organizations and their members to review and establish their own ethical frameworks and establish and operate a framework that is appropriate for their research purposes. Given the diverse nature of healthcare AI, the selection and explicit delineation of an appropriate ethical framework are crucial. The first stage supports engagement of a diverse array of experts and the public, including ethicists, legal scholars, patients, and laypersons to take an interest in the AI research process as necessary. Their collective input serves to establish guiding principles and rules crucial for the ethical conduct of research. This proactive approach aims to promote self-regulated ethical practices among researchers, distinct from mere compliance with legal regulations. Notably, the established ethical framework in stage 1 should be consistently referenced in most subsequent documentation.
- **Stage 2 **specifies plans for data collection and processing, mandating the creation of suitable datasets by designated data creator or “data curators” responsible for assembling and maintaining datasets (Leonelli 2016). The data collection and processing activities of researchers undergo to review by the Data Review Boards (DRBs). This board, established to oversight the ethical conduct of data-related procedures, evaluated data collection plan, anonymization methods, dataset size, quality, and management. The DRB operates within the research institution or as an independent body. Proposed by the Ministry of Health and Welfare of South Korea in the “Guidelines for Utilization of Healthcare Data,” the DRBs function as a committee of five or more individuals. Its responsibilities include assessing the suitability of processing pseudonymized information within an institution, reviewing the adequacy of pseudonymization, and managing the use of pseudonymized information within and outside the institution (Ministry of Health and Welfare of South Korea Dec 2022). This paper proposes that the DRB or a data appropriateness review entity comprising researchers, developers, and external members. This entity would review the data collection and management system before commencing healthcare AI research. Such proactive review aims to ensure the safety, appropriateness, feasibility, and absence of biases in data utilization for healthcare AI research.
- **Stage 3** involves the selection and preliminary assessment of algorithms, making the initiation of full-scale research. At this stage, researchers and developers undergo an IRB review encompassing all facets of conducting the study. They are required to provide extensive justifications concerning the study’s objectives, data standardization, and potential biases. The IRB, compared to the DRB, evaluates the appropriateness of the algorithm, the predictability and validity of results based on initial dataset, the reliability and safety of data management, and ensures the unbiased use of algorithm and data. Researchers, for reporting their conduct to the IRB, should consistently refer to the ethical framework established in stage 1. Considering that data utilization might vary concerning the algorithm used, distinct review rules are set by the DRBs and the IRBs. The former focuses on data management practice, while the latter oversees data utilization practices. This stage functions similar to phase I where the accuracy and appropriateness of the algorithm are determined and reviewed based on validated preliminary data. It can be paralleled with phase I safety assessments in clinical trials, wherein the interaction of an experimental medical device or drug with the human body is examined based on a small number of research subjects.
- **Stage 4 **encompasses the training, validation, and evaluation of the algorithm using the collected real-world data. The training data should be divided into train and test sets, and a pre-prepared validation set, distinct from the training data, is essential for validating healthcare AI algorithms to prevent overfitting and assess real-world applicability. The management of the validation process is imperative to avoid the exportation of models that are only useful during the training process to the actual application phase. and it is recommended that the research and development organization check this process. In the context of healthcare AI applications such as diagnostic imaging, patient risk prediction, and personalized treatment planning, each employing base algorithms ranging from deep learning to decision trees, the need for tailored validation processes becomes clear. For diagnostic imaging or patient risk prediction models, the validation process should primarily focus on rigorous statistical evaluation to ensure accuracy and reliability. Personalized treatment planning systems necessitate validation that emphasizes clinical relevance and the improvement of patient outcomes. These validation processes are essential for assessing the reliability of healthcare AI models. This stage can be seen as akin to phase II in clinical research, the phase that evaluates the effectiveness of a medical device or drug against a placebo. The emphasis is particularly placed on validating the trained algorithm and its relevance to clinical procedures.
- **Stage 5** involves the deployment of the developed healthcare AI algorithm into practical settings. The regulatory landscape governing healthcare AI implementation may vary based on its real-world application within a country. In South Korea, for example, AI model is evaluated and approved as a medical device by the Ministry of Food and Drug Safety. Moreover, for seeking for the National Health Insurance reimbursement, assessing safety, effectiveness, and economic evaluation from responsible regulators are mandatory. Throughout the step, the organization requires to pursue external validation for its development process, algorithms, and applications while prioritizing transparency. Furthermore, since the nature of healthcare AI includes continuous learning and development as part of its attributes, stage 5 also assigns responsibility for ongoing monitoring, identifying the entity accountable for managing the model. This stage corresponds to phase III, large trials, in clinical trials, where large-scale RCTs are used to determine the applicability of a treatment or new drug, in terms of determining the real-world applicability of a healthcare AI algorithm and putting it to work in the field.
- **Stage 6** mandates all parties involved to review the process of the continued deployment and ongoing development once the developed algorithm or model has been put into operation in a healthcare setting. Continuous review of use of the model and the functionality of the ethical framework remains pivotal. Maintaining transparent and collaborative communication among all stakeholders emerges as a necessity. In addition, vigilant monitoring of ongoing evolution of the model is imperative to prevent that decision-making based on real-world data might lead to unintended harms. This phase emphasized the follow-up and surveillance of algorithms and models post-launch, analogous to phase IV, post-market Surveillance in clinical research, which refers to the follow-up phase after clinical implementation of a medical device or drug.

The six-stage healthcare AI research governance proposed in this study can be compared to the five-phase standard, BS30440, recently proposed by the UK (Sujan et al. 2023). Set to take effect in the second quarter of 2023, BS30440 provides guidelines for validating AI systems in healthcare in the UK context. The guidelines reflect the product life-cycle of healthcare AI, which consists of inception, development, validation, deployment, and monitoring. Compared to the UK guidelines, the six stages presented in this paper add a preliminary ethical framework design and committee verification of data collection and management, distinguishing stages between algorithm determination and subsequent training, validation, and evaluation. BS30440 lacks stipulations for preliminary procedures or data management, integrates algorithm determination and training as a singular process, and makes validation as a separate process. Notably, our study’s governance procedure is designed to extend existing clinical research management procedures, whereas BS30440 establishes novel procedures. This study only examines these distinctions not to favour one framework over the other but to underscore the global development and application of similar governance procedures, extending beyond South Korea.

## Limitations and Future Research

The governance guidelines bear inherent limitations. Foremost, they do not decisively address the liability associated with possible harm resulting from healthcare AI applications. In the case of healthcare AI research and application involving multiple parties, it is necessary to examine whether the harm caused can be assumed the same as the existing medical liability process. For example, if a patient is physically harmed in the process of utilizing a healthcare AI device, but it turns out to be a problem with the algorithm rather than the fault of the medical practitioner or device user, who should be held liable?

Navigating liability questions amidst the overlapping influences of various actors poses challenges (Kim 2017). While the governance of healthcare AI research needs to address the issue of liability, it is limited by the fact that the guidelines in the study focus on proposing an ethical model grounded in self-regulation, addressing the intricacies of liability remains a significant challenge. Moreover, the procedures are set to be adjusted according to each country’s regulatory procedures, which is because the procedures correspond to existing clinical research guidelines, but it is necessary to examine whether they can be properly operated in real-life situations. This is an area that requires empirical verification by applying the guidelines to actual healthcare AI research governance. Therefore, this paper calls for further research on the healthcare AI governance guidelines presented here to address the issues identified above, especially linking it the legal standard to regulation.

To address the identified limitations, researchers are actively engaged in ongoing efforts in education of AI researchers and the public, social communication, and the revision of the guidelines. These initiatives will ensure a comprehensive societal understanding and adoption of healthcare AI research ethics, encourage researchers and developers to accept the need to conduct research ethically, and thereby facilitate the operationalization of ethical governance systems at both institutional and national levels within the South Korean context. As a result, these endeavours will significantly contribute to the establishment of a robust ethical normative framework for healthcare AI research in this country.

Since the governance settings presented in this study are from the perspective of a specific country, it is necessary to collect the opinions of researchers and bioethicists from other countries through international discussions and reviews. In order to facilitate such discussions, this study aims to inform other countries about the governance system established in South Korea and, using this study as a starting point, collect multi-perspective and multi-disciplinary views on healthcare AI research governance that have not yet been organized and provide basic data on the establishment of cross-border healthcare AI research governance.

## Conclusion

The aims of this study are to present a healthcare AI research governance system founded on the South Korean ‘Research Ethics Guidelines for AI Researchers in Healthcare’ and to elucidate each procedural step. The six-stage healthcare AI research governance framework mirrors the healthcare AI research and development process, and is designed in harmony with the existing clinical research management systems. This parallel structure facilitates the utilization of established research management resources and foster mutual understanding among researchers and institutions for conducting ethical research procedures. Nonetheless, the guidelines are likely to reflect the specificities of the Korean healthcare environment, emphasizing the need for further international dialogue and refinement.

## Data Availability

The framework employed in our research is included in the English version of “Research Ethics Guidelines for Healthcare AI Researchers” (KNIH 2023). This document is currently in the process of being published. Upon its publication, we will promptly provide the relevant link.

## Footnotes

1 Cross-Industry Standard Process for Data Mining

2 Knowledge Discovery in Database

3 Sample, Explore, Modify, Model, and Assess

4 Cognitive Project Management for AI
